# Peer-Mediated Intervention for the Development of Social Interaction Skills in High-Functioning Autism Spectrum Disorder: A Pilot Study

**DOI:** 10.3389/fpsyg.2016.01986

**Published:** 2016-12-23

**Authors:** Jairo Rodríguez-Medina, Luis J. Martín-Antón, Miguel A. Carbonero, Anastasio Ovejero

**Affiliations:** ^1^Center for Transdisciplinary Research in Education, University of ValladolidValladolid, Spain; ^2^Department of Psychology, Excellence Research Group GR179 Educational Psychology, University of ValladolidValladolid, Spain; ^3^Department of Psychology, University of ValladolidPalencia, Spain

**Keywords:** high-functioning autism spectrum disorder, inclusion, social skills, peer mediation, recess, elementary school

## Abstract

Autism Spectrum Disorder (ASD) is characterized by difficulties with social interaction and communication, which manifest at school especially in less structured situations such as recess. Recess provides opportunities for relationship with peers in a natural context, for which students with ASD may not be equipped with the necessary skills to use without support. Using a single-case design, we evaluated an intervention applied in recess to improve the social interaction skills of a student with high-functioning ASD mediated by his peers without ASD, in second grade of elementary school. This intervention includes different strategies to initiate the peers without ASD, using direct instruction, modeling, and social reinforcement carried out in the recess setting. After 14 sessions, changes were observed in the rates of initiating and responding to interactions, and a negative trend in the percentage of time that the student maintained low-intensity interactions or was alone. Teachers and family perceived improvements in social skills, more peer acceptance, and increase in the frequency and duration of social interactions. This intervention can help teachers to apply research-based practices to improve some social interaction skills in high-functioning students with autism in inclusive school environments.

## Introduction

Last revision of the Diagnostic and Statistical Manual of Mental Disorders (DSM-5; American Psychiatric Association, 2013) defines autism spectrum disorder (ASD) as a single spectrum disorder characterized by deficits in social communication and interaction, as well as restricted repetitive patterns of behavior, interests, or activities, observed in early childhood. However, for higher functioning individuals, these symptoms may not become fully manifest until social demands exceed limited capacities and therefore impair everyday functioning (American Psychiatric Association, 2013). Consequently, children with high-functioning autism spectrum disorder (HFASD) present certain communicative and social interaction characteristics, and challenging behavior that require specific attention from the teachers. An intervention adapted to their needs can improve not only aspects of communication and language but also aspects related to social skills and adaptive behavior (Barry et al., 2003; Reichow and Volkmar, 2010; Wong et al., 2015).

Social interaction with peers influences an individual’s development during childhood and adolescence and has an impact on academic, social, and emotional success, as well as on quality of life (Reichow et al., 2013). Social interaction is one of the main difficulties of students with ASD, which may affect their successful inclusion. Students with HFASD are more likely to engage with their peers without special educational needs (Bauminger et al., 2003). However, they have fewer friends and their friendships are of poorer quality (Kasari et al., 2011; Calder et al., 2013) and they have a worse perception of friendship, companionship, closeness, security, and help (Chamberlain et al., 2007; Solomon et al., 2011; Camargo et al., 2014). This usually leads to their isolation, even during recess and in inclusive educational settings (Anderson et al., 2004; Harper et al., 2008; Kasari et al., 2012), where a percentage of social interaction lower than 53% of the time would justify the need for a specific intervention to improve their social interactions (Shih et al., 2014). In this same line, Locke et al. (2016) compares the social behavior during recess of 51 students with ASD from seven schools with the behavior of classmates without autism. Their results indicate that students with autism spent approximately 30% of recess time alone, whereas their peers without autism were alone 9% of recess time, without differential effects as a function of the school.

It is essential to bear this in mind, because the development of their social skills is conditioned by their personal characteristics and by the socio-cultural practices of their settings (Ochs et al., 2004). Among the personal characteristics are the development of language, motor skills, learning style, social motivation, socio-emotional competence, or self-esteem (Ochs and Solomon, 2010), whereas the socio-cultural practices are related to values, attitudes, and interests that define the peer group’s culture (Corsaro, 1993; Conn, 2014, 2015), among which are included rules, codes, and the stability of the spatial arrangement of the environment. Gender differences are of particular importance (Torres et al., 2003; Werling and Geschwind, 2013; Dean et al., 2014; Anderson et al., 2016). For example, boys with autism in classrooms with more than 21 students undergo greater loss of social connectivity throughout of the school term than those in classrooms with smaller groups. The opposite effect is observed in girls with autism (Anderson et al., 2016).

Education of students with autism has been much influenced by a medical model, very focused on differences and deficits (Conn, 2016a), not sufficiently considering that peer group involvement can facilitate these individuals’ social integration at this formation stage and, at the same time, improve their classmates’ attitudes toward diversity (Koegel et al., 2011), an inclusive school environment seems appropriate for the development of socio-communicative skills in students with HFASD (De La Iglesia and Olivar, 2007; Conn, 2016b). This can help them to form a stable network of friends with whom to carry out activities even outside the school context, creating meaningful interpersonal ties that can be maintained over time (Koenig et al., 2009). Nonetheless, research yields diverse results (Owen-DeSchryver et al., 2008; Kasari et al., 2011). Some studies indicate that education in the regular classroom offers an appropriate environment for the acquisition, development, maintenance, and generalization of social interaction skills (Bauminger et al., 2003), whereas others state that it is insufficient, underlining that effective inclusion is unlikely to succeed unless a specific intervention, adapted to the characteristics of students with HFASD, is carried out (Ochs et al., 2001). Recently, many researchers agree that recess is an ideal inclusive context in which to practice social skills and communication (Harper et al., 2008; Owen-DeSchryver et al., 2008; Darretxe and Sepúlveda, 2011; Lang et al., 2011; Mason et al., 2014), because such skills occur in a natural context with greater possibilities of generalization and maintenance (Koegel et al., 2012a). Therefore, we must take into account the resources offered by the physical environment to facilitate interaction (Schoen and Bullard, 2002; Yuill et al., 2007; Couper et al., 2013) and where we can use some strategies like delimitation of play areas by painting playground markings, the use of students’ chalk drawings and the texts they wrote down in their journal notebooks.

Peer-mediated intervention has been identified as an effective procedure for the learning of social skills (Wang H.T. et al., 2011; Wang S.Y. et al., 2011; Kamps et al., 2015), with positive effects on academic, personal, and social development: (a) it increases communication among students with and without autism, reducing maladaptive behaviors (Lee et al., 2007), (b) it increases the possibility of interaction with peers, reducing demands for teacher attention (Chan et al., 2009); (c) it encourages practicing diverse social skills with a variety of classmates, increasing the possibilities of generalization to other contexts (Watkins et al., 2015); (d) it can be incorporated into the regular school setting and is more effective to improve social skills than individualized work carried out by support professionals (Kasari et al., 2012); and (e) teaching strategies to peers so they will interact with classmates with ASD increases the opportunities of socialization in natural contexts (Wong et al., 2015). These benefits are higher in inclusive school environments (Chan et al., 2009; Camargo et al., 2016), where other classmates’ participation allows practicing social skills in natural contexts (Rogers, 2000). This provides better outcomes regarding the creation of social networks, friendship quality, and solitude reduction than other intervention models that do not involve peers (Locke et al., 2012).

Several reviews of research that employed this model have found a high percentage of effectiveness (Watkins et al., 2015; Whalon et al., 2015), although with different intensities. Chan et al. (2009) found positive results in 91% of the 42 studies reviewed, with students aged 2–13 years, although these authors advise interpreting the results with caution, given the limitations in the fidelity of the implementation, as classmates, rather than professionals, applied the intervention.

Chung et al. (2007) conducted a study to assess the improvement of social skills in four students with HFASD, aged 6–7 years, with a 12-week intervention. For this purpose, they used an observation code adapted from Thiemann and Goldstein (2001) in 10-min sessions of free play, before and after the intervention. The results indicated an increase in the frequency of initiating conversations, as well as appropriate responses to peer interaction in three of the four students.

Kasari et al. (2012) compared two intervention models for the development of social skills in students with HFASD, one peer-mediated and the other with individualized professional support, in 20-min sessions implemented at recess, twice a week for 6 weeks (a total of 12 sessions). To evaluate its effectiveness, they used sociometric procedures, observational records, and questionnaires of social skills as perceived by teachers. Their results indicated that the time the children were alone at recess was significantly reduced only in the students who participated in the peer-mediated intervention, and the effects of this intervention persisted 3 months later without additional support. In addition, these students received more preference nominations, although this increase was not reciprocal in students with autism.

The results of several investigations report that a peer-mediated intervention at school may increase the frequency and duration of social interaction in inclusive school environments during recess. McFadden et al. (2014), with four students aged 5–8 with ASD, three with HFASD, applied the teacher’s direct instruction combined with the peer-mediated intervention for 7 months. The intervention included social skills sessions before recess for the entire class, peer reinforcement, adult feedback during recess, and a token economy system. The results showed an increase of initiation and response behaviors to classmates by students with ASD, as well as an increase in the frequency of peers’ communicative actions toward students with ASD. Similar results were found by Koegel et al. (2012b) with students aged 5–6, and by Owen-DeSchryver et al. (2008) with two students aged 7 and 10 years.

Finally, it should be taken into account that there are some obstacles to this intervention model, among which are (Locke et al., 2015): (a) the number and availability of school teachers; (b) the professionals’ lack of training (Prino et al., 2016); (c) the organization of recess; (d) the prioritization of academic goals; and (e) the availability of resources. To this is added the little time available for instruction of social skills (Owen-DeSchryver et al., 2008). There is scarce research using a collaborative approach on the efficacy of trainings that engage the entire class, together with teachers who are not specialized in special education and the family, even in inclusive environments. These are aspects that this study aims to address.

### Aims of the Present Study

This study draws on the practical evidence showing that children with HFASD can benefit from the opportunities provided at recess to acquire and practice certain social skills through peer-mediation, and contributes to the improvement of our knowledge of these intervention models. Interventions are more effective in elementary education (Reichow and Volkmar, 2010), and we agree with Locke et al. (2016) in assessing social interaction during recess to determine whether children with ASD are unengaged, because solitary is not the same as solitude. Many children with ASD would like to have friends and do not wish to be alone (Bauminger and Kasari, 2000). Accordingly, the following goals were proposed: (a) to design and implement an intervention to improve the social interaction skills of a second-grade elementary education student with HFASD, during recess; (b) to assess the effectiveness of this intervention in terms of frequency, duration, and quality of initiations and responses to social interactions during recess, as well as their perception by his family and teachers, and in other contexts; (c) to analyze the changes in peer ratings after the intervention, and (d) to analyze changes in their social interaction skills. We hypothesize that a peer-guided intervention, applied during recess: (a) will increase the frequency and duration of social interaction, (b) will reduce the time that the boy is alone, (c) will increase his acceptance by peers, and (d) will increase his social interaction skills.

## Materials and Methods

### Participants and Instructors

A high-functioning student with autism, aged 8 years and 3 months, and his 16 classmates (eight males) from the second-grade classroom of Spanish public school were participants. Those participants had the normative age corresponding to this grade (8 years), none of them had autism, and two were from another country (Morocco). Target student was in the regular classroom for the entire school day.

He was initially diagnosed with Asperger’s syndrome, and he has been enrolled in the same regular school since early childhood education. He obtained a composite score of 120 on the Wechsler Scale for Preschool and Primary Scale of Intelligence, 3^rd^ edition (WPPSI-III), which places him at percentile 91; he also obtained an average-high score on the Peabody Picture Vocabulary Test (PPVT-III), which places him at percentile 55. The Childhood Autism Spectrum Test (CAST, formerly called the Childhood Asperger Screening Test; Scott et al., 2002) indicates that he has difficulties with peer relationships, he rarely approaches other children to play, he does not consider it important to fit in his peer group, and he generally does not show the same interests.

This is a Spanish public school located in a medium-low socio-cultural neighborhood, which provides education to nearly 300 students from pre-school and elementary education, of whom 15% come from other countries (mainly Bulgaria, Romania, and Morocco). The observations and intervention were carried out in the schoolyard the 30-min recess (12:00–12:30 pm). During recess, the first- and second-grade students go out to playground area that is assigned to them. This area is 30 m by 25 m, enclosed by the school building: one part is covered by a porch, while the other part has a row of trees on one side and a clear central area. These elements are the only ones where the students can play (mainly to hide). There are no other alternative spaces in which to play.

The present study was conducted in accordance with the 1964 Helsinki declaration and its later amendments or comparable ethical standards, and the approval of the Provincial Offices of Education, Department of Education of the Autonomous Government of Castilla y León (Spain). Participation in the study was voluntary. All subjects gave written informed consent in accordance with the Declaration of Helsinki.

### Measures

#### Observation of Social Interaction during Recess

We designed an observation register with the following categories: (a) frequency of initiation of social interaction, (b) response frequency to a social interaction, (c) frequency of challenging interactions, (d) percentage of time that he is alone, and (e) amount of time he interacts adequately (the student participates actively in an activity with one or more classmates) and inadequately (the student shows hostility or anger toward one or more classmates while participating in an activity). This instrument was developed after an initial exploratory phase, drawing from proposals like the Behavior Coding Scheme (BCS; Hauck et al., 1995), or the Playground Observation of Peer Engagement (POPE; Kasari et al., 2012). **Table 1** shows the categories, recording units, and their definitions, which were recorded from the beginning to the end of the session (continuous recording). The Obansys software (Mangold International, 2012) was used for coding. It allows recording of specific behaviors (e.g., answering or initiating an interaction) and actions that extend over time (e.g., being alone, interacting adequately) in real time.

**Table 1:** Categories of observation codes of interaction in recess.

Categories	Description
**Interaction mode**	
Initiates an interaction	The student adequately starts a verbal, non-verbal, or mixed social interaction with one or more classmates; it is distinguished from the continuation of the prior social sequence because it involves a change in the recipient (in a group, he is talking to a classmate and addresses a different one; a change in activity, or in the reference).
Responds to interaction	The student responds adequately to a direct verbal or non-verbal interaction of one or more classmates, which is distinguished from the continuation of the previous social sequence by a change in the classmates to whom he responds or in the activity. There is a clear communicative intention.
Challenging interaction	The student initiates or responds inappropriately to an interaction with one or more of his classmates.
**Type of interaction**	
He is alone	The student is alone, without doing any activity or he performs some activity at a distance of more than 1.50 m from his classmates.
Adequate interaction	The student participates actively in an activity with one or more classmates. They share a game, collaborate in an activity, talk, laugh, etc.
Inadequate interaction	The student shows hostility or anger toward one or more classmates while participating in an activity.
Low intensity interaction	Proximity without communicative intention. The student remains next to or closely follows (<1.5 m) a classmate or group of classmates, either without participating in a particular activity or as a mere observer.

#### Peer Rating

This procedure evaluates a student’s average peer acceptance. Students are given a list with the names of all their classmates, and are asked to rate them, answering the question, *How much do you like to play with...?* on a three point scale (*very much, not much, not at all*). This method is more accurate than those based on peer nomination because it ensures that all the students are rated in a weighted manner by all their classmates (Monjas et al., 2014), and it presents adequate psychometric properties (Asher and Dodge, 1986; Cillessen, 2009). We chose a three-point scale because at this age, they might have difficulties being accurate with more response options. This difficulty is greater in students with autism, for whom two- or three-point response scales are recommended (Conn, 2016a), or, at least, the use of numbers and colors associated with each scale option (Buron and Curtis, 2012).

We considered two of the indices provided by this procedure: the status index (*I*_s_), which refers to the number of ratings received, and the expansiveness index (*I*_e_), with the emitted ratings. In addition, we computed: (a) the number of reciprocal preference ratings, when they nominated each other mutually as *very much*; (b) the number of reciprocal rejection ratings, when they nominated each other mutually as *not at all*; (c) the number of opposite ratings, when one student rated the other as *very much* and the other rated the former as *not at all* or vice versa; and (d) the absence of ratings.

#### Cuestionario de Habilidades de Interacción Social (CHIS; Monjas, 2002, in English, Social Interaction Skills Questionnaire)

This questionnaire has 60 items distributed in six subscales of different social interaction skills: (a) Basic Social Skills, (b) Skills to Make Friends, (c) Conversational Skills, (d) Skills Related to Emotions and Feelings, (e) Interpersonal Problem-solving Skills, and (f) Skills in Relationships with Adults. The family or teachers rate the frequency of each behavior on a 5-point Likert-type scale (*never, hardly ever, several times, almost always, always*). The questionnaires were completed by the family and the teachers. It is a useful tool to design interventions, as it identifies the child’s deficient or problematic skills (Monjas, 2002). Although there is no information about its psychometric properties, this questionnaire is widely used in the educational setting (see, for example, Postigo et al., 2012).

#### Social Validity

The student recorded his degree of satisfaction with the activities in his *recess agenda* with the help of his family, rating it on a five-point Likert-type scale (from *very bad* to *very good*) with numeric and visual support. There was also an activity of weekly reflection in which he had to indicate in which situations he felt happy, sad, angry, and/or scared during recess; in which games he participated; and the number of classmates with whom he did so. To determine the teacher and the specialists’ degree of satisfaction with the implementation of the intervention, they completed a brief satisfaction survey. It contains six items (see supplemental data) adapted from the proposal of Mason et al. (2014), rated a 5-point Likert-type scale (*strongly disagree* to *strongly agree*). There is also a space to express general comments or an opinion about the intervention. The family is also asked to express their opinion of the intervention.

### Design

This is a single-case design (SCD, Kennedy, 2005) using observational methodology, using the visual analysis of graphic data displays, a method commonly used to verify the efficacy of interventions to increase prosocial or academic behavior (Horner et al., 2005). Observational methodology is usually applied in combination with other data collection methods and in multiple designs, but its greatest benefit is found in studies outside the laboratory, as it maximizes ecological validity (Bakeman and Gnisci, 2006; Volkmar, 2011). Following the recommendations of Conn (2016a), systematic observation was complemented with participatory observations: appraisals by the involved professionals, the family, and also by all the involved students because the experiences that each student had to transcribe of the interviews of their classmates allowed us to know their rating of the intervention procedure, in a natural way and without adult influence.

### Procedure

After obtaining permission from the school and the educational authorities, as well as the informed consent of the families, we began the systematic observation. The recordings were carried out entirely at recess during the four phases of the study. To ensure homogeneity between observation sessions, the recording started daily at 12:20 h and concluded at the sound of the siren that signaled the end of recess. Like Locke et al. (2016), the last 10 min of recess were chosen because they provide more stable data because they are not conditioned by incidents that delay going out to recess; also, in the first part of the recess, the students eat their snacks, and there are few and very variable social interactions. We observed every day of the week, except if the target student was absent, or for some reason the students did not go out to the playground. All the sessions were simultaneously recorded by two observers from beginning to end, without interruption. The two observers remained at a distance of approximately 3–6 m from the student, so that they could accurately record his activity and comments. When he was alone and far from his classmates, the observers increased their distance to avoid excessive focus and possible stigmatization of the student.

The intervention was mainly guided by the regular teacher, after having been trained by special education professionals. In the initial and final assessments, other specialist teachers and the family were also involved. The observers were special education professionals who were not employed by the school and who were trained in the interface of the recording software, the observation categories, and their definitions, with actual use in three trial sessions, in conditions similar to those they would experience during the study. Furthermore, before and after each session of the exploratory phase, we carried out a review and feedback among the observers to deepen the definition of the categories and units of observation, and to share meanings, in order to ensure the correct use of the recording tool. The observers had collaborated with the school in the diverse activities as of the previous school term, so their presence at recess was habitual and did not surprise the students.

The study was conducted in four phases, as follows.

#### First Phase: Exploratory Phase

We designed the observation instrument, defining the categories, guaranteeing its between-observer reliability and usefulness in the specific application context. We conducted a total of nine initial observations for 2 weeks, applying the designed observation registry to verify whether all the displayed behaviors were significantly reflected. We also observed the students’ play preferences on the playground. The family and teachers filled in the questionnaire of social interaction skills.

#### Second Phase: Baseline

To calculate the necessary time for this phase, we used the method proposed by Gelfand and Hartmann (1968), which takes into account the variability of the behavior: 3 + [10 (HR-LR)/HR], where HR and LR are, respectively, the highest and lowest rate of the behavior during the first 3 recording days. This procedure yielded 14 days for the behavior with the greatest variability (*percentage of time that the student is alone at recess*). These rates were calculated by dividing the total frequency of occurrence by the total observation time in minutes, and they indicate the number of social initiations the student carries out per minute. These 14 observation sessions were conducted over 3 weeks.

#### Third Phase: Intervention

We designed an intervention program with a duration of 14 sessions, carried out over 4 weeks. It falls within the framework of the theory of the social learning of Bandura (1977), in which socially competent peers can model and appropriately reinforce their classmates’ socially skilled behaviors. All the students of the classroom, including the target student, participated similarly, which improves aspects of social interaction that are more difficult for students with HFASD, but it can also benefit the rest of the classmates. The first session was carried out in the classroom, lasting approximately 40 min, and had several goals. The first aim was to justify the presence of the observers during recess. The second was to explain the goal of the program, *The Recess Pals*, which, basically, is to improve the negative aspects identified at recess. The third goal was to differentiate between appropriate and inappropriate behavior at recess, providing various amusing alternatives, which follow a basic outline and are proposed in the form of a challenge: (a) seek a playmate or playmates, (b) propose a game, (c) set the rules, and (d) play in teams. For this purpose, we used a combination of strategies of peer initiation, reinforcement, and proximity which include direct instruction, modeling, and social reinforcement. Fourthly, we requested their voluntary cooperation to participate, which was obtained from all the students in the group. Finally, all the students completed the peer rating.

The second intervention session, *Recess Reporters*, is designed to familiarize the students with their classmates’ play preferences, and to practice the proposed skills. In pairs, they had to choose a classmate to interview, showing interest in his/her favorite games and play preferences during recess. For this purpose, they used a pencil-and-paper *journalist’s notebook*, which set out the specific steps to perform the activity. The students had to reach an agreement with their teammate about which classmate to interview, approach to an appropriate speaking distance, greet the classmate, presenting themselves politely and agreeably, and finally, start, maintain, and end a conversation, showing interest in the interviewee’s play preferences. The peer who was paired with the target student modeled the behaviors. The rest of the sessions were ludic, conducted entirely in recess, lasting approximately 20 min. After forming groups of free play, the students had to practice the previously proposed skills under the teacher’s supervision. In all the sessions, the students made suggestions and they sometimes included the preferences of their classmate with HFASD. The teacher allowed the students to select the games, and constantly reinforced the participants’ adequate behaviors, and when they were uncertain about what to play, the teacher suggested activities related to the preferences of the target student, but at the same time, integrating other interests of the rest of the classmates. Some strategies were also used to explain and understand the game rules, such as the delimitation of play areas and the use of students’ chalk drawings and the texts they wrote down in their journal notebooks.

After the last session, we applied the peer rating in the classroom. In this case, two students were absent.

#### Fourth Phase: Maintenance

This was conducted immediately after the intervention phase, recording 10 sessions for 3 weeks, the social interactions of the target student, but completely eliminating all the reinforcements.

### Data Analysis

To ensure the reliability of the observations of each behavior, we calculated the Intraclass Correlation Coefficient (ICC) and alpha coefficient of Krippendorf (KALPHA) of the data obtained by the two observers. For the ICC, we used a 95% confidence interval, and its values were interpreted according to the scale proposed by Landis and Koch (1977), in which values below 0.40 represent low reliability, values between 0.41 and 0.80 represent moderate to good reliability, and values above 0.81 are considered excellent reliability. To calculate KALPHA, we used the syntax developed by Hayes and Krippendorff (2007), where scores below 0.70 are considered to tend toward low statistical significance (Krippendorff, 2004). Both coefficients were calculated with SPSS, version 23, obtaining satisfactory rates of agreement, with ICC values between 0.79 and 0.99, and KALPHA values between 0.78 and 0.98.

We used the method of visual analysis of graphic displays of data to study changes in the rate and frequency of social interaction behaviors. This type of analysis takes into account the trend, level, and stability of data assessed within and between conditions (Lane and Gast, 2014). We also calculated the Non-overlap of All Pairs (NAP, Parker and Vannest, 2009), which measures the percentage by which two stages of observations differ, adopting values between 50% (no difference) and 100% (completely different). Up to 65%, the change is weak, from 67 to 92%, it is medium, and if it exceeds 92%, it is high.

Non-parametric Wilcoxon matched pairs test was used to determine possible differences in the peer rating and in the social interaction skills between the two assessment times. To estimate the effect size, we calculated the *r* statistic (Rosenthal, 1991, 1994), interpreting the results according to Cohen’s (1988) criterion, where 0.1 is a small effect, 0.3 is a medium effect, and 0.5 is a large effect.

## Results

### Social Interaction

**Table 2** presents the descriptive statistics of the rates of *initiation* and *response* to social interactions and *challenging interactions* during the four phases. These rates are calculated by dividing the total frequency of occurrence by the total observation time in minutes. The visual analysis of graphic displays of data indicates that, at baseline, the student had difficulties to initiate social interactions with his peers. Sometimes he approached or followed a classmate or group of classmates without addressing them directly and without any apparent communicative intention. When he wanted to call the attention of his peers on the playground, he sometimes used strategies like pushing or kicking. He did not usually initiate interactions when his classmates were playing games that he did not like.

**Table 2:** Descriptive statistics of the rate of behavior per minute in each situation in the study phases.

	Baseline^a^		Intervention^b^		Maintenance^c^	
Behavior	*M*	*SD*	*M*	*SD*	*M*	*SD*
Initiation of social interactions	0.27	0.20	0.31	0.23	0.57	0.37
Response to social interactions	0.17	0.15	0.28	0.25	0.51	0.21
Rate of challenging	0.31	0.24	0.13	0.25	0.09	0.16

*^a^*n* = 14, ^b^*n* = 14, ^c^*n* = 10.*

**FIGURE 1:**
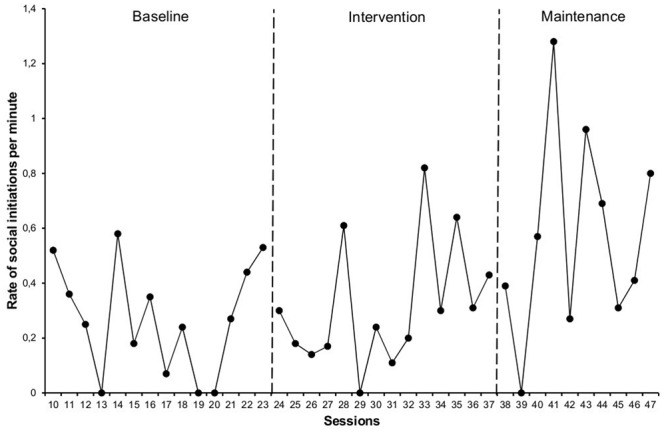
**Rate of social initiations per minute by the target student toward his typically developing peers**.

As shown in **Figure 1**, there was a slight increase between *the mean rate of social initiations* per minute between the baseline, at 0.27 initiations per minute (0.00–0.58), and the intervention phase, 0.31 (0.00–0.82). However, there was a greater increase at maintenance, 0.57 (0.00–1.28). There was a medium combined change between phases (NAP = 66%), and we highlight the change between baseline and maintenance (NAP = 76%) and between intervention and maintenance (NAP = 73%).

**FIGURE 2:**
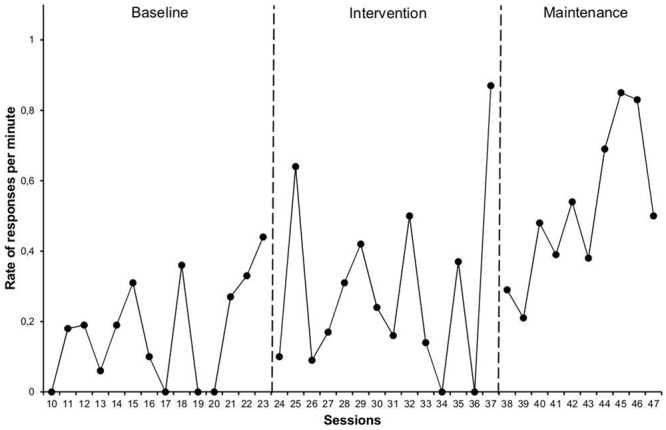
**Rate of responses per minute by the target student toward his typically developing peers**.

A similar effect was observed in the evolution of the *rate of responses to peer interactions* (**Figure 2**), with a slight increase in the mean rate of responses between baseline, 0.17 (0.00–0.44), and the intervention phase, 0.28 (0.00, 0.87), which was much higher at the maintenance phase, 0.51 (0.21–0.85). In this case, there was a medium effect (NAP = 76%) among the three phases. The most significant changes occurred between the baseline and maintenance (NAP = 92%) and between intervention and maintenance (NAP = 76%).

**FIGURE 3:**
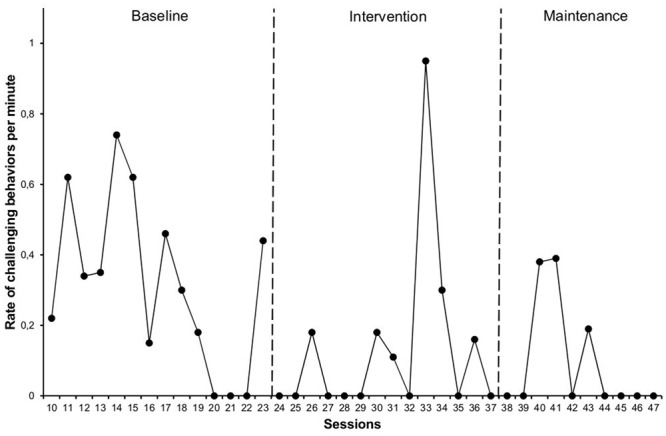
**Rate of challenging behaviors per minute**.

With regard to the *rate of challenging interactions*, we observed a progressive decrease between the different phases (**Figure 3**) although it was more pronounced between baseline, 0.31 (0.00–0.74), and the intervention phase, 0.13 (0.00–0.95), than in the maintenance phase, 0.09 (0.00–0.39). The change between phases was medium (NAP = 69%), with the most intense changes occurring between baseline and intervention (NAP = 75%) and between baseline and maintenance (NAP = 73%).

Although in general, the rate of behaviors such as kicking, pushing or insulting his classmates decreased, it was precisely during the intervention phase when the session with the highest rate of challenging interactions occurred. We believe that, in many cases, they were unfortunate attempts to start a social interaction with peers. However, we also observed situations in which the student may have had trouble understanding the context and non-verbal language, which led him to misinterpret his classmates’ gestures.

The student’s pattern of social interaction usually started with his being alone (15 min) while he ate his snack. He would sometimes follow the group from a distance of about 3 or 4 m (*low-intensity interaction*), or he would run around alone in the portico area (*is alone*), performing some stereotypy (*flapping*), or walking slightly on tiptoe. Subsequently, if no classmate initiated an interaction, he would always try to seek the same classmate, with whom he had a close relationship (this classmate had the highest acceptance rate of the group). However, he often had trouble addressing him verbally, so he would use physical interaction, such as grabbing, pushing, touching, etc. If he established the interaction, it was usually positive. However, if the classmate was playing some other game or was with another group, and he asked our participant to join them, the interaction tended to be low level, such as following them more closely, or watching them but without participating actively in the game. Usually, these low level interactions ultimately led to his being alone again.

**FIGURE 4:**
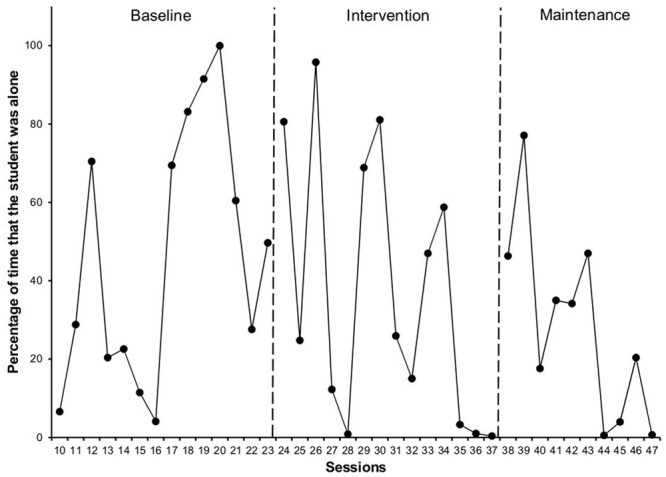
**Percentage of time that the target student was alone in each of the phases**.

The percentage of Time the Student is Alone during recess (**Figure 4**) decreased between baseline (*M* = 46.15, *SD* = 32.9) and the intervention phase (*M* = 36.82, *SD* = 34.3) by 10%, and during the maintenance phase (*M* = 28.27, *SD* = 24.6) by another 9%.

**FIGURE 5:**
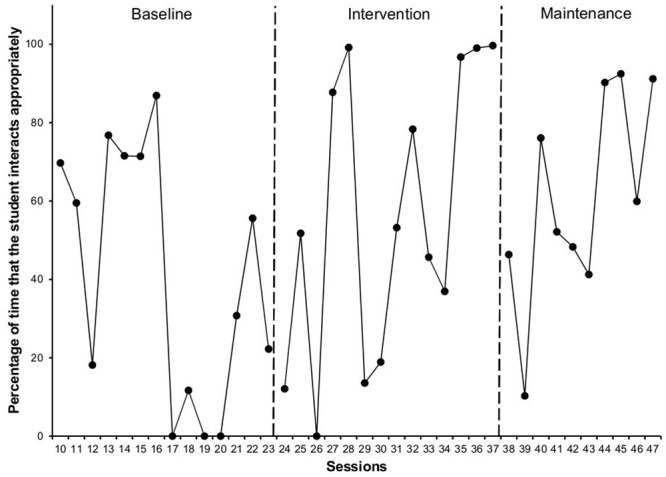
**Percentage of time that the target student interacts appropriately in each of the phases**.

With regard to the Time the Student Interacts Adequately with his peers (**Figure 5**), we observed a 15% increase between the baseline (*M* = 41.02, *SD* = 32.2) and intervention phases (*M* = 56.62, *SD* = 36.7), and a 4% increase in the maintenance phase (*M* = 60.81, *SD* = 26.7).

However, the percentage of Time the Student Interacts Inadequately with peers was very low, as the highest value he reached throughout the entire study was 5.2%, and this was stable throughout all phases. The average time that the student maintained a *low-intensity interaction* with his classmates was 12% at baseline, and it decreased to 6% in the intervention phase, again increasing to 11% in the maintenance phase.

### Peer Rating

In the procedure conducted before the intervention, the target student had an acceptance rate of *I*_a_ = 0.92 (range of -1 to +1), which indicated he was the second highest student in group acceptance. Moreover, only one classmate rated his preference for him as a playmate as *not much*; and four classmates did not give him any rating. After the intervention, he received practically the same mean rating, *I*_a_ = 0.93, which meant he was still among three most preferred students to play with and, similarly, only one classmate rated his preference for him as a playmate as *not much*. In this case, there were no missing ratings, as the two students who did not rate him previously were not present during the procedure.

Regarding the ratings issued (expansiveness), the target student rated his preference for five classmates negatively (*not at all*) and for six students as *not much*; he did not rate two of his classmates at all, and he only rated his preference as *very much* for three classmates, among them two girls. These last three ratings were reciprocal (these three classmates also rated their preference for him as *very much*). This was the lowest expansiveness index of the group, *I*_e_ = -0.14. Moreover, he gave opposite ratings to two classmates who rated him positively. However, this index was much higher after the intervention, *I*_e_ = 0.81, because he rated his preference for 13 of his classmates as *very much*, and 12 of these ratings were reciprocal; he rated his preference for three classmates as *not much*; and did not rate anyone as *not at all*. This change is statistically significant, *z* = -2.92, *p* = 0.004, with a large effect size, *r* = 0.78.

### Social Interaction Skills

The Wilcoxon signed-ranks test yielded a significant increase in the total number of social interaction skills between pre- and post-intervention (*M* = 144.50, *SD* = 32.88 vs. *M* = 169.75, *SD* = 30.40), with a large effect size, *z* = -5.48, *p <* 0.001, *r* = 0.70. This increase was also significant for each of the skills, except for the Relationship with Adults (**Table 3**). The effect size was high in all of them, although the highest was found in Basic Skills (*r* = 0.90) and in Interpersonal Problem-solving (*r* = 0.83).

**Table 3:** Differences in the Social Interaction Skills Questionnaire Scores before and after the Intervention.

	Pre-test		Post-test				
Social Interaction Skills Subscales	*M*	*SD*	*M*	*SD*	*z*	*p*	*r*
Basic Social Skills	23.25	7.14	29.25	4.92	-2.83	0.005	0.90
Skills to Make Friends	23.00	6.58	26.50	4.80	-2.26	0.024	0.71
Conversational Skills	26.00	9.02	29.00	6.06	-2.58	0.010	0.81
Skills Related to Emotions and Feelings	26.50	5.69	27.75	4.92	-2.36	0.018	0.75
Interpersonal Problem-solving Skills	21.25	4.27	25.50	4.65	-2.64	0.008	0.83
Skills in Relationships with Adults	29.50	5.45	31.75	6.45	-0.85	0.395	0.26
Total	144.50	32.88	169.75	30.40	-5.48	0.000	0.70

### Social Validity

During baseline phase, the student said he was pleased during recess when he “played with someone.” During intervention, he was content during recess, and he began to say that he was pleased “when he played with his friends.” In the maintenance phase, he was sad when “we were still playing and the siren sounded.” This evolution between phases may be related to the increase of the time in which the student interacted appropriately with his classmates. It should be noted that at baseline, he repeated that he was sad when “they ignore me” and when they “play something else”; and angry when “they play something else.” However, during the intervention, he reported that he was only sad when “they play something else,” but he also added that “I did not want to play because that game bored me.”

Of the four teachers who completed the satisfaction survey, two of them agreed that the intervention improved the participants’ social interaction skills, while the other teachers strongly agreed. Three of the teachers strongly agreed that the students had enjoyed themselves and were sufficiently motivated. The classroom teacher pointed out that the students “have learned to relate more, they play better, and there is more camaraderie.” “They liked it very much and they were interested,” and the target student “was very interested in the program.”

The family indicated that, before the intervention, their child tended not to tell them anything about school and he did not talk much about recess. However, after the intervention, he tended to comment anecdotes or events that occurred during the day, and talk about recess more frequently, and he showed more interest in playing with his classmates. For example, the family reported that “he tells us whether or not his classmates wanted to play with him, how he felt, and even how he convinced them to join his game.” They consider that the fact of having enjoyed himself with his classmates has led him to have expectations of success in establishing relationships, and even to take the lead in seeking others to play with.

## Discussion

This study examines the effectiveness of a peer-mediated intervention program for the development of social interaction skills, in a student with HFASD. Changes were observed in all the variables, maintaining a positive trend in the rates of initiating and responding to interactions, and a negative trend in the percentage of time that the student maintained low-intensity interactions or was alone.

These results are similar to those obtained by Owen-DeSchryver et al. (2008), Banda and Hart (2010), Banda et al. (2010), Mason et al. (2014), and McFadden et al. (2014) and indicate that the intervention had a positive effect, which is also consistent with other studies using the same methodology (Whalon et al., 2015), and many peers without autism who participated, compared with the two or three classmates who are usually selected in this type of interventions (Banda et al., 2010; Kasari et al., 2012). It is true that a greater number of peers may cause a great variability of socially skillful behaviors to be modeled. However, this variability is present in the social environment of the school population, which can lead to a greater generalization of the learnings with other peers (Gresham, 1998) than when there is only one peer per student with ASD (Pierce and Schreibman, 1995).

Regarding the rates of interaction initiation, the results are similar to those obtained by Owen-DeSchryver et al. (2008), showing a slight increase in the intervention phase. Concerning the delay in the change of level, McMahon et al. (2013) note that interactions with classmates other than the usual ones in the early stages of the intervention may be a factor of stress and anxiety, which is eliminated in the final stages. Although we introduced the participant’s play interests and preferences, this technique was shown to be more effective as the intervention advanced and his peers suggested the games, which may contribute to explain this delay. With regard to the time the student interacts adequately, he started with an initial percentage of 41%, lower than the cut-point of 53% established by Shih et al. (2014), which indicated that a specific intervention was necessary. After the intervention, that percentage was exceeded (57%) and even increased slightly in the maintenance phase (61%). We also underline the 18% reduction of the time that the target student spent alone, decreasing from 46% of the time at baseline to 28% during the maintenance phase, a percentage similar to that found in the study of Locke et al. (2016).

Regarding the total duration, some recess interventions have obtained positive and statistically significant results with 12 to 16 sessions, with a duration of between one and a half to 3 months (Kasari et al., 2012; Kretzmann et al., 2015). However, like Goldstein et al. (2007), taking into account the results obtained in maintenance phase, we consider that it may be more appropriate to extend the intervention over a longer period of time, spacing out the sessions in order to reduce the risk of tiring the students.

As regards the peer rating, the target student received one of the highest ratings of the classroom, so it was not possible to improve his social peer acceptance, as would have been expected. However, if we relate the results of the systematic observation, like Kasari et al. (2011), we find little relationship between the number of nominations received by the student and real social interaction on the playground. Despite receiving a high peer rating, at baseline, the target student was still alone, or was not chosen among the first to form teams or pairs in games. It is encouraging that, in contrast to the findings of Kasari et al. (2012), there was a significant change in the student’s ratings of his classmates after the intervention, as the number of classmates with whom he liked to play increased, as did the number of reciprocal preference ratings, and no classmates were rated as rejected by him.

It seems clear that the intervention has globally helped to improve this student’s social interaction skills, especially the basic skills and the interpersonal problem-solving skills (Barry et al., 2003; Kasari et al., 2012). Hence, he has more strategies to handle any social situation and to deal with conflictive situations, which tend to be one of the most significant difficulties for people with ASD. In contrast, there was no change in the interaction skills with adults, which was expected because the intervention focused on peer mediation and at a time—recess—when the adult plays a minor role.

Finally, the success of any intervention program depends on the level of satisfaction of the people involved, especially of the student with HFASD, as well as on their belief in its benefits and application possibilities. Something similar should occur in the family environment if generalization is to be ensured. From the student’s statements, we can deduce an improvement in his perception of interpersonal relations and social motivation but also in his capacity to decide not to participate if he is not interested in a game. We consider, therefore, that the intervention has guaranteed an environment that respects his autonomy, dignity, and individuality. The teachers and the family were also highly satisfied, with results similar to those found by Mason et al. (2014) and McFadden et al. (2014), perceiving adequate participation and engagement of all the involved parties, in addition to improvement in the interpersonal relationships and school motivation of target student.

### Conclusion, Limitations, and Future Directions

This work has revealed that an inclusive, peer-mediated intervention during recess improved the student’s social skills as perceived by his teachers and family, his peer acceptance, and the frequency and duration of his social interactions. This intervention has the advantage of involving all the students of the class, the non-specialist teachers, and the family.

However, as it is a pilot study, the intervention was only implemented in a single group, which obviously has limitations for its validity. It is necessary to verify its effectiveness in more subjects, in different grades, and in different schools. It is also recommended to observe the possible evolution at the beginning of a new school course, although doubtless, new variables such as the new course itself, different classmates, and a new recess area would affect the possibility of establishing relationships among the outcomes. Also, it would have been adequate to verify whether the intervention improved the social climate of the classroom, also modifying the relations and status of the classmates without autism. Our impression is that this did occur because, after the intervention, there was a global increase of reciprocal preference ratings. However, we cannot determine whether this increase manifests in more frequent, longer lasting, and better quality social relations because that would require a systematic observation of all the students and all the interactions. In future works, we recommend the use of audiovisual means to record and subsequently analyze these situations. On another hand, the intervention began without having established a stable baseline, and there was large intraphase variability. Although this is a common problem in this type of research (Thiemann and Goldstein, 2004; Harper et al., 2008; Koegel et al., 2009), the results should be interpreted with caution. In the future, it would be necessary to control some factors that may affect this variability, such as the student’s fatigue, situations or conflicts in the classroom prior to recess, type of game, or groupings.

We consider that this intervention proposal can help and encourage teachers to apply research-based practices to improve some social interaction skills in students with HFASD in inclusive school environments. It is not an easy task but it is possible. There are important obstacles to its application, such as the varying number of students per classroom, the training of the non-specialist teaching personnel, the insufficient number of specialist teachers in regular schools, or the pressures of the socio-cultural environment and the families for education to focus on academic performance, derived from the educational policies. However, the regular teacher is the main reference for all the students in the class and the one who, ultimately, can facilitate the application of educational strategies that promote an inclusive environment, always with the advice of specialists of special education. Consequently, this type of interventions can encourage the use of the schools’ personal resources, taking an ecological perspective of educational attention. We must be aware that the intervention proposal presented herein is not suitable for all students with ASD. Depending on each case, specific, individualized interventions that are fully implemented by specialists may be more effective. However, and to conclude, the purpose is to have a repertory of educational strategies that can be applied if they are considered to facilitate the integral development of the autistic child.

## Ethics statement

The present study was conducted in compliance with ethical standards of the institutional research committee and with the 1964 Helsinki declaration and its later amendments or comparable ethical standards. Permission was obtained from the school and the educational authorities, as well as the informed consent of the families. Informed consent was obtained from all individual participants included in the study.

## Author Contributions

JR-M conceived, designed and led the study, performed the measurement and participated in drafting the manuscript. LM-A designed and led the study, participated in analysis and interpretation of the data, coordinated data collection, and participated in drafting the manuscript. MC and AO contributed to the study design and coordination and helped to draft the manuscript. All authors approved the final manuscript as submitted.

## Conflict of Interest Statement

The authors declare that the research was conducted in the absence of any commercial or financial relationships that could be construed as a potential conflict of interest.
