# Supplementary material for: Peer-Mediated Intervention for the Development of Social Interaction Skills in High-Functioning Autism Spectrum Disorder: A Pilot Study
Source: Front Psychol. 2016 Dec 23;7:1986. doi: 10.3389/fpsyg.2016.01986 (PMC5179565; doi:10.3389/fpsyg.2016.01986)
Supplement: Supplementary file 1 [file Data_Sheet_1.docx]

**Appendix**

Teachers' Assessment of the Intervention

| 1. The intervention seems to have improved the social interaction skills of the participating students.  2. The students who participated in the intervention have enjoyed themselves and were sufficiently motivated.  3. The goals, intervention techniques, and results of the program were satisfactory both for the recipients and for the teachers and the family involved.  4. Beneficial effects of the program that were not initially anticipated were observed.  5. The resources (human, material, organizational, and time and space) that the program required are consistent with the results achieved.  6. There is evidence of the positive impact of the program on other aspects or members of the school and/or context. |
| --- |

*Note*. Adapted from Mason, R., Kamps, D., Turcotte, A., Cox, S., Feldmiller, S., and Miller, T. (2014). Peer mediation to increase communication and interaction at recess for students with autism spectrum disorders. *Res. Autism Spectr. Disord.* 8, 334-344. doi:10.1016/j.rasd.2013.12.014
